# Self management and patient understanding of diabetes in the older person

**DOI:** 10.1111/j.1464-5491.2010.03142.x

**Published:** 2010-12-19

**Authors:** J Hewitt, L Smeeth, N Chaturvedi, C J Bulpitt, A E Fletcher

**Affiliations:** † London School of Hygiene and Tropical Medicine and Portsmouth Hospitals TrustollegeLondon, UK; ‡ Queen Alexandra HospitalPortsmouth; * Imperial CollegeLondon, UK

**Keywords:** cognitive impairment, elderly, self management, understanding

## Abstract

**Abstract:**

**Aim:**

To examine knowledge and management of diabetes by older people.

**Methods:**

A representative sample of 1047 people with Type 2 diabetes, aged 75 years and over, were asked a series of questions relating to their diabetes management and their understanding of self management. The impact of cognitive impairment and socio-economic status were assessed.

**Results:**

The majority of people, 1015 (96.9%), were under the care of a health professional and 1018 (97.2%) were taking insulin, tablets, controlling their diet or a combination. Cognitive impairment (Mini-Mental State Examination ≤ 23) was found in 235 (22.5%) people. Recent eye, foot and dietician assessment was reported by 813 (77.7%), 836 (79.7%) and 326 (31.1%) people, respectively. A quarter overall and 70% of those taking insulin tested their blood glucose. In the insulin group, 78 (54.2%) reported hypoglycaemia and those with cognitive impairment gave more incorrect responses when asked about diabetes management. Socio-economic status made very little difference to any of these outcomes.

**Conclusions:**

Most older people with diabetes, regardless of their socio-economic status, are under the care of a healthcare professional and use medication or diet to manage their disease. Large numbers also attend foot and eye examinations. However, over one fifth of older people with diabetes have cognitive impairment. Older people had a reasonable understanding of their diabetes management but this was worse in those people with cognitive impairment.

Diabet. Med. 28, 117–122 (2011)

## Introduction

In modern diabetic management, patients are often encouraged to take responsibility for aspects of their own care. There are several components of successful long-term patient-based management. These include self-medication, monitoring blood glucose, annual review, input from dietetic services and regular screening for foot and eye problems [1–3]. These different management strategies are often conducted by a number of different healthcare professionals, either alone or in combination. What effect sole or joint care has on older people with diabetes is not known.

As complication risk increases with diabetes duration, optimization of self-care strategies in older individuals, where risks are likely to be high, is particularly important. In order to achieve these goals, older people with diabetes need a good understanding of their condition and to be sufficiently intact cognitively and motivated to make the appropriate decisions regarding their health.

In this paper, we report the results of a questionnaire aimed to examine the different types and understanding of diabetic management in older people with diabetes from a range of socio-economic backgrounds. We also examine the effect of cognitive impairment on our results.

## Patients and methods

The study from which the data in this paper derive was a factorial cluster randomized trial designed to evaluate different methods of assessment and management of older people. The trial design and results have been previously been described [4,5]. In brief, 106 general practices from the MRC General Practice Research Framework, selected to be representative of the UK distribution of mortality and deprivation (Jarman scores), were randomized to a Universal or Targeted health assessment, with further randomization to management by a primary care team or a hospital-based geriatric team. In the Targeted arm, participants underwent a brief assessment and formed the control group for the trial. The diabetes information in this group was limited and they were not considered further in the study described here. Universal arm participants underwent an in-depth assessment by a practice nurse, which covered a wide range of health and social problems, including a biochemical screen. Participants were assessed for cognitive function using the 30-point Mini-Mental State Examination (MMSE) [6]. A score of 23 or below was taken to indicate cognitive impairment. An assessment of socio-economic status was made using the Carstairs index [7,8]. We used any one of the following criteria to categorize a person as having diagnosed Type 2 diabetes: a positive response to the question ‘Have you ever been told by a doctor that you have sugar diabetes?’; currently taking glucose-lowering medication; and a diagnosis of diabetes recorded in the participant’s computerized general practice medical record. These patients were additionally asked questions relating to knowledge and understanding of diabetes. An interview with a proxy (usually a carer) was conducted in a small number of people who were unable to complete the interview alone.

All patients on the general practitioner list aged 75 years and over who were not resident in nursing homes were eligible for the study. Data for this study were collected between 1995 and the end of 1998.

Tables 1 and 2 show the questions asked to the participants with diabetes.

**Table 1:** Questions of diabetic understanding asked to all participants with diabetes

Who do you normally see for your diabetes? (can be more than one response)	Family doctor/general practitioner, hospital doctor, practice/district nurse, no one
What treatment are you on for your diabetes? (can be more than one response)	Diet alone, tablets, insulin injections, no treatment
Do you test your blood for sugar?	Yes or no
If yes, how often?	Approximately once a day or less than weekly
In the last year, have you had your feet examined?	Yes or no
In the last year, have you had your eyes examined?	Yes or no
In the last year, have you discussed your diet with a dietician?	Yes or no

**Table 2:** Questions of diabetic understanding asked only to people taking insulin

Have you ever had a low blood sugar or ‘hypo’?	Yes, no or don’t know
If you have a low blood sugar, should you increase your diabetes treatment?	Yes, no or don’t know
If you have a low blood sugar, should you take a sugary drink or snack?	Yes, no or don’t know
If you have the flu, should you stop taking your insulin?	Yes, no or don’t know

## Results

Of 21 710 people invited to participate in the study, 15 286 people (response rate, 70.4%) aged 75 years and over attended. Of these, 15 095 people provided adequate information to ascertain a diagnosis of diabetes. A total of 1047 (6.9%) people who had a clinical diagnosis of diabetes mellitus were identified [9]. There were 489 (46.7%) men and 558 (53.3%) women. The average age of the people with diabetes was 80.9 years (range 75–100 years) and included 54 people aged over 90 years. There were 235 (22.5%) people with diabetes and an MMSE ≤ 23, compared with 2874 (20.7%) people with an MMSE ≤ 23 in the population without diabetes (*P* < 0.001). Within the population with diabetes, the distribution of people according to quintiles of Carstairs index was: 178 (17.0%), 250 (23.9%), 237 (22.6%), 198 (18.9%), 98 (9.3%), least to most deprived, with 86 (8.2%) missing responses for this variable.

### Source of medical advice

The majority of people, 1015 (96.9%) from a total of 1047, were under the sole or joint care of a healthcare professional. Approximately two-thirds were seen by a general practitioner and, of these, a third were also seen by a hospital doctor (Table 3). Approximately half had contact with a nurse in primary care, the majority in combination with a general practitioner or hospital doctor. Also listed in the table are people who were under the sole care of their general practitioner, a hospital doctor or a primary care nurse. Overall, 485 people (46.3%) were under sole care only and 530 (50.6%) were under joint diabetic care of some description. A small proportion (*n* = 32, 3.1%) reported that they saw no one for their diabetes management.

**Table 3:** Medical supervision of participants with diabetes

	General practitioner (GP)	GP only	Hospital doctor	Hospital doctor only	Nurse	Nurse only	GP and hospital doctor	GP and nurse	Hospital doctor and nurse	GP, hospital doctor and nurse	No one
Who do you normally see about your diabetes? (Can be more than one) *n* = 1047 (missing = 0)	655 (62.6%)	229 (21.9%)	239 (22.8%)	101 (9.6%)	569 (54.4%)	155 (14.8%)	104 (9.9%)	379 (36.2%)	92 (8.8%)	57 (5.4%)	32 (3.1%)

Deprivation, measured by the Carstairs index, showed that the most deprived people were less likely to see their general practitioner (*P* < 0.001), but no less likely to see a hospital doctor (*P* = 0.13), nurse (*P* = 0.37) or no one (*P* = 0.51). The most deprived sections of the population were also more likely to be under sole diabetic care (*P* < 0.001).

### Treatment regimes

Approximately one third of people with diabetes were managed by diet alone, approximately half were on oral medication, usually on its own, and just over 10% were taking insulin, again as the sole glucose-lowering agent. Diet control was mentioned as part of the treatment package by 439 (41.9%) of the study population. (Table 4) The use of insulin was not affected by age and none of these results varied between men and women. There were 12 people who saw no one for their diabetes and took no treatment. All these people had self reported their diabetes diagnosis.

**Table 4:** Treatment regimes for participants with diabetes

	Diet only	Tablets	Insulin	Diet and tablets	Diet and insulin	Tablets and insulin	Diet, tablets and insulin	No treatment
What treatment are you on for your diabetes? (Can be more than one) *n* = 1047 (missing 0)	373 (35.6%)	533 (50.9%)	144 (13.8%)	63 (6.0%)	2 (0.2%)	32 (3.1%)	1 (0.1%)	27 (2.6%)

### Use of diabetic services

Overall, 813/1047 (77.7%) of those with diabetes reported they had undergone an eye examination within the last year. There were 836 (79.9%) who reported a foot examination and 326 people(31.1%) had seen a dietician. Attendance at these services was not affected by social status, as measured by the Carstairs index (*P* = 0.31, *P* = 0.05 and *P* = 0.16 for eye, feet and dietician, respectively). There were 729/1047 (69.6%) people who reported both foot and eye examination and 263/1047 (25.1%) people reported they had had both examinations and seen a dietician within the last 12 months.

When comparing people under joint diabetic care with those under sole care, both eye and foot examinations were more likely in the joint-care groups, but not dietician attendance (88.4 vs. 80.2%, *P* < 0.001 for eyes; 89.5 vs. 83.7%, *P* = 0.03 for feet; 35.6 vs. 32.8%, *P* = 0.61 for dietician attendance).

### Frequency of home glucose testing

In total 247/1047 (23.6%) people reported that they tested their blood glucose at home (75/1047, 7.2%, had a missing response for this question). People who tested were younger (80.2 years compared with 81.1 years, *P* = 0.003), but there was no gender difference. Of the group who tested their blood, 97 (39.3%) reported they tested daily and 138 (55.9%) weekly or less frequently (12 people had missing data for this response). There were 50 (20.2%) people with cognitive impairment who tested their blood glucose compared with 177/725 (24.4%) with cognitive impairment who did not test (*P* = 0.1). Social status did not affect the likelihood of home testing (*P* = 0.15) or the frequency of testing (*P* = 0.05). Likewise, being under sole or joint care of diabetes did not affect the likelihood of home testing (*P* = 0.57) or its frequency (*P* = 0.28).

Considering the 144 individuals taking insulin, the proportion and frequency of glucose testing was higher than the overall population with diabetes (*P* < 0.001 for each). There were 101/144 (70.1%) people testing their blood glucose. Of these, 65/101 (64.4%) people tested approximately once per day and 36 (35.6%) measured it weekly or less. Age (*P* = 0.96) and sex (*P* = 0.77) were unrelated to the frequency of testing.

### Hypoglycaemia and individual understanding of diabetes management among people using insulin

The majority of people managing their diabetes with insulin correctly identified the steps to be taken in the event of low blood sugar, although individuals with cognitive impairment, who formed approximately one quarter of this group, were significantly more likely not to know what to do (Table 5). Approximately one quarter of participants did not know whether and how medication should be altered in the face of acute ill health; again, those with poor cognitive function fared significantly worse.

**Table 5:** Understanding of diabetes management in older participants with diabetes taking insulin: with and without cognitive impairment

	Total number of people taking insulin (%)	Number of people taking insulin who were cognitively intact (MMSE ≥ 24) (%)	Number of people taking insulin who had cognitive impairment (%)	*P*-value	
				Comparing people with and without cognitive impairment	
	*n* = 144	*n* = 107	*n* = 37	Age and sex adjusted	
Have you ever had a low blood sugar or ‘hypo’?	Yes	78 (54.2)	63 (58.9)	15 (40.5)	*P* = 0.08 (comparing yes/no answers only)
	No	55 (38.2)	37 (34.6)	18 (48.7)	
	Don’t know	9 (6.2)	5 (4.6)	4 (10.8)	
	Missing	2 (1.4)	2 (1.9)	0	
If you have a low blood sugar, should you take a sugary drink or snack?	Yes	125 (86.8)	100 (93.5)	25 (67.6)	*P* = 0.013 (comparing correct and incorrect responses)
	No	5 (3.5)	3 (2.8)	2 (5.4)	
	Don’t know	13 (9.0)	4 (3.7)	9 (24.3)	
	Missing	1 (0.7)	0	1 (2.7)	
If you have a low blood sugar, should you increase your diabetes treatment?	No	100 (69.5)	83 (77.6)	17 (46.0)	*P* = 0.008 (comparing correct and incorrect responses)
	Yes	13 (9.0)	8 (7.5)	5 (13.5)	
	Don’t know	30 (20.8)	16 (14.9)	14 (37.8)	
	Missing	1 (0.7)	0	1 (2.7)	
If you have the flu, should you stop taking your diabetes tablets or insulin?	No	108 (75.0)	88 (82.3)	20 (54.1)	*P* = 0.017 (comparing correct and incorrect responses)
	Yes	4 (2.8)	4 (3.7)	0	
	Don’t know	31 (21.5)	15 (14.0)	16 (43.2)	
	Missing	1 (0.7)	0	1 (2.7)	

MMSE, Mini-Mental State Examination.

Social deprivation made no difference to the frequency of hypoglycaemia (*P* = 0.97), increasing diabetes treatment (*P* = 038) or sick-day management (*P* = 0.55). Similarly, sole or joint diabetic management did not affect the responses for any of these questions (hypoglycaemia *P* = 0.55, taking a snack *P* = 0.12, increasing treatment *P* = 0.29 or sick-day management *P* = 0.99). However, people from lower social economic groups gave more incorrect responses regarding taking a snack in the presence of hypoglycaemia (*P* = 0.003).

## Discussion

In this large community-based study of older people, aged 75 years and above, approximately 7% had previously diagnosed diabetes. The majority were under the sole or joint care of their general practitioner and approximately half were managed with oral medication. Provision of annual retinal and foot examination was high, with over three quarters of participants obtaining these, especially people under joint models of diabetic care. However, only a third had review by a dietician in the previous year and only a quarter tested their blood glucose at home. In those on insulin, approximately one quarter had evidence of cognitive impairment and this significantly reduced their ability to understand the actions required in the event of a low blood sugar or acute infection.

Only 3.0% of participants were not seeing a medical professional of any description for their diabetes, much lower than a previous survey estimate of 100 UK patients aged at least 65 years, which found 19% of patients had no medical supervision [10]. Less than 3% of our study population reported taking no treatment whatsoever. Therefore, the vast majority of elderly people appear to be under treatment in the community, either alone or using a combination of diabetic treatment regimes, with insulin continuing to be used into the extremes of old age. Diet was listed as a treatment (either alone or in combination) in over 40% of people, implying that these individuals understand that diet formed part of their diabetic management. Perhaps, more important is the converse perspective; well over half did not consider that diet formed part of their diabetic management.

The National Service Framework (NSF) for diabetes [3] recommends the use of diabetic specialist nurses, dieticians, eye specialists and chiropodists. They add to the overall quality of care and increase patient knowledge [2,11–17]. Previous estimates in institutionally based elderly populations have suggested eye examination to occur in between 52 and 72% of older people each year [10,18]. A high proportion of older community-dwelling people with diabetes underwent regular eye and foot examination, but less than one third saw a dietician. It is difficult to determine if these results reflect increased awareness of these services, especially those recommended for yearly attendance, in people with diabetes or simply reflect the usage of these services in older people regardless of diabetes status. Our figures reflect favourably when compared with the 2009 Trovino study, which showed that being aged over 75 years was directly associated with a lower level and frequency of diabetic care [19]. The only direct comparison available from their study showed that 15% of their older population had had an eye examination within the last year.

Both older age and diabetes itself are risk factors for cognitive impairment [20–22]. Therefore, in older people with diabetes, individual understanding of the management of diabetes is likely to be complicated by poor cognition. Our study confirmed the high prevalence of cognitive impairment. The prevalence was high in the population as a whole, in those who tested their blood glucose and in those who were taking insulin. This suggests that cognitive impairment was high among older people with diabetes who have the capacity for hypoglycaemia and that home testing may be unreliable.

The potential benefits (or otherwise) of home glucose testing are disputed [23–27]; nonetheless, individuals, young and old, are still currently taught how to perform home glucose testing. For glucose testing to be effective, it is presumed that it should be performed regularly. In keeping with previous studies in younger people, we found the frequency of blood testing to be less than daily in over half our population and the frequency of testing to decrease with age [28,29]. For example, in the Kaiser Permanente Population in Northern California, 67% of people with Type 2 diabetes tested their blood glucose less than daily [28]. However, in people who tested their glucose, the actual frequency of testing was not affected by age. In the individuals taking insulin, the degree and frequency of testing was higher, perhaps reflecting the increased efforts by health professionals to encourage glucose testing.

All patients with diabetes taking hypoglycaemic medication need to be aware of the symptoms of hypoglycaemia, how it arises, how to prevent it and how to treat it. Older people are particularly susceptible to hypoglycaemia and often are not aware of the symptoms [30]. In our study, nearly 70% of people taking insulin tested their blood glucose, with over half reporting hypoglycaemia. Encouragingly, the majority of these people knew that they should take a sugary snack or drink in the presence of a low blood sugar. Of more concern, were the figures reflecting treatment options in the presence of hypoglycaemia and ‘sick-day’ management, where over a quarter gave incorrect responses. While our figures suggest that most older people with diabetes understand some basic principles of diabetic care, further education of some aspects of diabetes management may be helpful. These figures were worse in people with cognitive impairment and identification of these individuals should be recommended. Two limitations of these data should be highlighted. Firstly, our study did not record whether participants had ever received diabetic education of any sort and, secondly, some of the sub-group analysis involved small numbers of participants.

Numerous studies have assessed different care models and methods of diabetic education. Three Cochrane reviews of this area have shown that some benefit can be gained from joint diabetic control [31–33]. However, the effect of any of these interventions on diabetic outcomes has not been proven. Our study showed that older people with diabetes under joint care were more likely to have an annual eye and foot examination. Whether this will make any difference to diabetic eye and foot disease in the longer term remains to be seen and further randomized controlled trials should be recommended.

Worsening social status is known to affect the health of all age groups. Encouragingly in our study, it appeared to have less effect. This may reflect an improved level of health provision for these people. It is also possible that the Carstairs index, which has been independently validated and is widely used, was not able to detect any difference that did in fact exist.

This study showed that the majority of older people with diabetes, regardless of socio-economic status, saw a medical professional, underwent some form of treatment and the majority understood basic diabetes management. Our results provide a summary of community-based provision for elderly people with diabetes, an area noted for lack of evidence [2]. However, the model of diabetic care employed, either joint or sole care, seemed to have only limited benefits within our population of people with diabetes. Data for this study were collected between 1995 and 1998, before the introduction of the National Service Framework for diabetes, and it will be particularly interesting to see if these figures change as the National Service Framework continues its development and implementation. The level of understanding of hypoglycaemia and its management in this population was high, but could be improved. In addition, there was a high prevalence of cognitive impairment throughout the whole population of people with diabetes, including those at risk of hypoglycaemia. Identification of older people with diabetes with cognitive impairment should be recommended.

## Competing interests

Nothing to declare.

## Abbreviations

MMSE: Mini-Mental State Examination
